# Optimal Path Choice in Railway Passenger Travel Network Based on Residual Train Capacity

**DOI:** 10.1155/2014/153949

**Published:** 2014-07-06

**Authors:** Fei Dou, Kai Yan, Yakun Huang, Li Wang, Limin Jia

**Affiliations:** ^1^School of Traffic and Transportation, Beijing Jiaotong University, Beijing 100044, China; ^2^State Key Laboratory of Rail Traffic Control and Safety, Beijing Jiaotong University, Beijing 100044, China

## Abstract

Passenger's optimal path choice is one of the prominent research topics in the field of railway passenger transport organization. More and more different train types are available, increasing path choices from departure to destination for travelers are unstoppable. However, travelers cannot avoid being confused when they hope to choose a perfect travel plan based on various travel time and cost constraints before departure. In this study, railway passenger travel network is constructed based on train timetable. Both the generalized cost function we developed and the residual train capacity are considered to be the foundation of path searching procedure. The railway passenger travel network topology is analyzed based on residual train capacity. Considering the total travel time, the total travel cost, and the total number of passengers, we propose an optimal path searching algorithm based on residual train capacity in railway passenger travel network. Finally, the rationale of the railway passenger travel network and the optimal path generation algorithm are verified positively by case study.

## 1. Introduction

Railway as a kind of large volume passenger transportation modes has been developing in China and other countries (e.g., USA and Europe) in an even larger scale, and it plays a role of the passenger transportation systems. How to make the passenger service decision making more demand responsive has stood under the spotlight. Therefore, as a base of the railway passenger service decision making, it is of paramount importance to study the railway passenger path choice.

Generally, it is assumed that railway passengers will choose their paths prior to their departure and then strictly follow their paths till they reach their destinations. More and more different train types are available for travelers; many paths need to be chosen by travelers from their departure to their destination. However, most travelers could not choose accurately depending on their travel demand before departure. Thus, special attention should be paid to the generalized cost of different paths in modeling railway passenger's optimal path choice, which serves as a useful planning tool at both strategic and operational levels.

Rational railway passenger travel network is backbone of passenger optimal path choice. In most previous studies, a schedule-based transfer network was constructed by three kinds of nodes, which are station nodes, train arrival nodes, train departure nodes, and five kinds of arcs, which are boarding arcs, alighting arcs, running arcs, stopping arcs, and transferring arcs. And they defined the generalized cost function depends on a variety of contributing factors, including three items: travel time, fare, and discomfort cost (see [1]).

In one of the most influential papers on schedule-based transit modeling, Tong and Richardson [2] argued that a development algorithm for finding the time-dependent minimum path between two stations in a schedule-based transit system. Afterwards they proposed a recursive algorithm for finding the reasonable paths in a transit network (see [3]) and proposed a specially developed branch and bound algorithm, which was used to generate the time-dependent minimum path for passenger flow assignment. The assignment procedure was conducted over a period in which both passenger demand and train headway are varying. They presented an overview of the research that had been carried out to develop the schedule-based transit assignment model (see [4, 5]). In addition, more schedule-based path-finding algorithms were presented for the transit network, such as forward-search algorithm and backward-search algorithm (see [6–8]). However, from empirical studies it appears clear that the vehicle capacity constraints were not considered. Thus, in order to have a more realistic description of technological progress, the concept of strategies in schedule-based transit networks with capacity constraints was extended in a study (see [9, 10]).

As it is described above, railway passengers will choose the path by the minimum generalized cost prior to their departure and then strictly follow their paths till they reach their destinations. Generally, a passenger will not transfer to another train in travel, if the previous train is congested or exceedingly uncomfortable. Our aim is to determine the shortest/optimal paths and depict them to the passenger for decision making before the journey. The developed algorithm based on residual train capacity does not inform the passenger which path is congested in travel; however, it can provide the passenger choices to make a decision on which path to traverse for completing his/her journey.

In the transportation field, many researchers presented the various path searching algorithms for different types of networks to solve shortest paths problem [11–14]. In [15] a multicriteria shortest path searching approach was presented based on the classical shortest path problem on a network representing the urban multimodal transportation system, to aim at minimizing the overall cost, time, and users' discommodity associated with the required paths. Authors in [16] presented a new route set generation algorithm based on shortest path search with link elimination. The proposed procedure combines a Breadth First Search with a topologically equivalent network reduction and ensures a high diversity between the routes. They demonstrated the usability of the algorithm, whose performance and resulting route sets were compared with those of a stochastic choice set generation algorithm.

The method widely applied in shortest path searching problem is Dijkstra's algorithm (see [17–19]). Recent studies have focused on optimized Dijkstra's algorithm combined with other methods to search the shortest path. Pradhan and Mahinthakumar used Floyd-Warshall algorithm and Dijkstra's algorithm to search the all-pairs shortest path in a large-scale transportation network [20]. Szűcs used the Dempster-Shafer theory and Dijkstra's algorithm to search the best route planning in a transport network where the network type can be arbitrary: a network of bus routes, a network of tram rails, a road network, or any other type of a transport network [21]. Namkoong et al. presented the optimal path searching algorithm which was basically based on Dijkstra's algorithm and the tree-building algorithm used to construct optimal paths in developing route guidance systems and traffic control systems [22]. In [23, 24] the methods is based on multicosts constraints and time-window constraints, followed by the application of a *K* shortest path algorithm to find out the first *K* shortest paths in a schedule-based transit network. According to previous studies, we proposed an optimal path generation algorithm of railway passenger travel network based on residual train capacity and Dijkstra's algorithm to solve the optimal path searching problem.

The paper is organized as follows. In the next section, we construct the railway passenger travel network and present the notations used in the development of this network. The generalized cost function and residual capacity of the path as the foundation of path searching procedure are developed, and network topology is analyzed. The construction method of the railway passenger travel network on the basis of the train timetable is proposed. In Section 3, the optimal paths searching algorithm based on residual train capacity is presented. An illustrative example is presented to demonstrate the validity of the passenger travel network construction method and the optimal path searching algorithm in Section 4. In the last section, we present the conclusions and recommendations for future research.

## 2. Railway Passenger Travel Network Construction

### 2.1. Notation

We have the following notations

 
*v*_*city*,*tr*_^*s*,*ar*^ or *v*_*city*,*tr*_^*s*,*de*^: the node, which includes attributes as follow: station (*s*), node type (*ar*/*de*), city (*city*), and train number (*tr*),
 
*cid*: the code of* city*,
 
*sid*: the code of station *s*,
 
*tid*: the code of train* tr*,
 
*vtype*: the type of node, which consists of arrival node and departure node,
 
* vtime*: time of the train arrival node *v*_*city*,*tr*_^*s*,*ar*^ or time of the train departure node *v*_*city*,*tr*_^*s*,*de*^,
 
*E*_1_, *E*_2_, *E*_3_, *E*_4_, *E*_5_: the set of boarding arcs, the set of running arcs, the set of stopping arcs, the set of transferring arcs, and the set of alighting arcs, respectively,
 
*V*_1_: the set of the origin node *v*_*O*_ and the destination node *v*_*D*_,
 
*V*_2_: the set of the train arrival nodes and train departure nodes,
 (*v*_*O*_, *v*_*city*,*tr*_^*s*,*de*^) ∈ *E*_1_: the arc between the origin node *v*_*O*_ and the train departure node *v*_*city*,*tr*_^*s*,*de*^ for the train* tr* at station *s* in city,
 (*v*_*city*1,*tr*_^*s*,*de*^, *v*_*city*2,*tr*_^*s*,*ar*^) ∈ *E*_2_: the arc between the train departure node *v*_*city*1,*tr*_^*s*,*de*^ for the train* tr* at station *s* in* city 1* and the train arrival node *v*_*city*2,*tr*_^*s*,*ar*^ for the train* tr* at station *s* in* city 2*,
 (*v*_*city*,*tr*_^*s*,*ar*^, *v*_*city*,*tr*_^*s*,*de*^) ∈ *E*_3_: the arc between the train arrival node *v*_*city*,*tr*_^*s*,*ar*^ for the train* tr* at station *s* in* city 1* and the train departure node *v*_*city*,*tr*_^*s*,*de*^ for the train* tr* at station *s* in* city 2*,
 (*v*_*city*,*tr*1_^*s*,*ar*^, *v*_*city*,*tr*2_^*s*′,*de*^) ∈ *E*_4_: the arc between the train arrival node *v*_*city*,*tr*1_^*s*,*ar*^ of the train* tr1* and the train departure node *v*_*city*,*tr*2_^*s*′,*de*^ of the transfer train* tr2* at station *s* in city,
 (*v*_*city*,*tr*_^*s*,*ar*^, *v*_*D*_) ∈ *E*_5_: the arc between the train arrival node *v*_*city*,*tr*_^*s*,*ar*^ for the train* tr* at station *s* in city and the destination node *v*_*D*_,
 
*t*_arc_: the travel time of the arc,
 
*f*_arc_: the travel fare of the arc,
 
*eid*: code of the arc,
 
*T*[*i*][*j*]: the travel time from node *i* to node *j*,
 
*N*[*i*][*j*]: residual train capacity from node *i* to node *j*,
 
*F*[*i*][*j*]: the travel fare from node *i* to node *j*,
 
*C*[*i*][*j*]: the generalized cost from node *i* to node *j*,
 Maxnum: a maximum value.

### 2.2. Railway Passenger Travel Network

**Figure 1:**
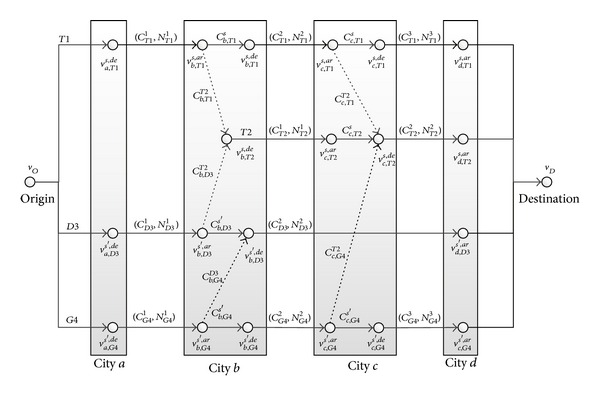
Railway passenger travel network.

Railway passenger travel network *G*(*V*, *E*) is constructed based on the schedule-based transfer network by two kinds of nodes and five kinds of arcs, where *V* and *E* are the set of nodes and the set of arcs, respectively. Define the set of nodes as *V* = *V*_1_ ∪ *V*_2_ and the set of arcs as *E* = *E*_1_ ∪ *E*_2_ ∪ *E*_3_ ∪ *E*_4_ ∪ *E*_5_. There are two kinds of nodes in this network, one kind of nodes is transition node which is original node *v*_*O*_ or destination node *v*_*D*_. The other kind of nodes is train arrival nodes or train departure nodes. There are five kinds of arcs in this network: the boarding arcs, running arcs, stopping arcs, transferring arcs, and alighting arcs. To illustrate, Figure 1 presents a railway passenger travel network with four cities and four trains. Clearly, a passenger travel path from the origin node to the destination node includes a series of boarding, running, stopping, transferring, and alighting arcs in the passenger travel network (*V*, *E*).

**Figure 2:**
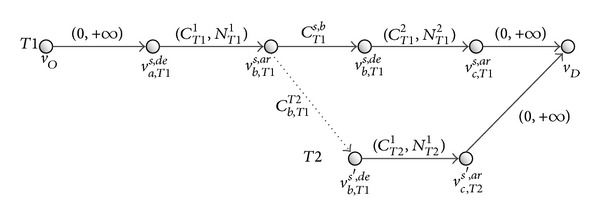
Two kinds of nodes and five kinds of arcs in railway passenger travel network.

In Figure 2, there are two paths which are* P1* and* P2* in the passenger travel network with three cities (city *a*, city *b*, and city *c*) and two trains (train* T1* and train* T2*). Path* P1* = {(*v*_*O*_, *v*_*a*,*T*1_^*s*,*de*^), (*v*_*a*,*T*1_^*s*,*de*^, *v*_*b*,*T*1_^*s*,*ar*^), (*v*_*b*,*T*1_^*s*,*ar*^, *v*_*b*,*T*1_^*s*,*de*^), (*v*_*b*,*T*1_^*s*,*de*^, *v*_*c*,*T*1_^*s*,*ar*^), (*v*_*c*,*T*1_^*s*,*ar*^, *v*_*D*_)}, which includes the boarding arc (*v*_*O*_, *v*_*a*,*T*1_^*s*,*de*^), running arcs (*v*_*a*,*T*1_^*s*,*de*^, *v*_*b*,*T*1_^*s*,*ar*^)∪(*v*_*b*,*T*1_^*s*,*de*^, *v*_*c*,*T*1_^*s*,*ar*^), stopping arc (*v*_*b*,*T*1_^*s*,*ar*^, *v*_*b*,*T*1_^*s*,*de*^), and alighting arc (*v*_*c*,*T*1_^*s*,*ar*^, *v*_*D*_). Path *P*2 = {(*v*_*O*_, *v*_*a*,*T*1_^*s*,*de*^), (*v*_*a*,*T*1_^*s*,*de*^, *v*_*b*,*T*1_^*s*,*ar*^), (*v*_*b*,*T*1_^*s*,*ar*^, *v*_*b*,*T*2_^*s*′,*de*^), (*v*_*b*,*T*2_^*s*′,*de*^, *v*_*c*,*T*2_^*s*′,*ar*^), (*v*_*c*,*T*2_^*s*′,*ar*^, *v*_*D*_)}, which includes the boarding arc (*v*_*O*_, *v*_*a*,*T*1_^*s*,*de*^), running arcs (*v*_*a*,*T*1_^*s*,*de*^, *v*_*b*,*T*1_^*s*,*ar*^)∪(*v*_*b*,*T*2_^*s*′,*de*^, *v*_*c*,*T*2_^*s*′,*ar*^), transferring arc (*v*_*b*,*T*1_^*s*,*ar*^, *v*_*b*,*T*2_^*s*′,*de*^), and alighting arc (*v*_*c*,*T*2_^*s*′,*ar*^, *v*_*D*_).

### 2.3. The Generalized Cost Function

As it is described in Section 1, a passenger will not transfer to another train in travel, even if the previous train is congested or exceedingly uncomfortable. Our aim is to determine the shortest/optimal paths and depict them to the passenger for decision making before the journey. Therefore, the most important factors of passenger travel are the travel time and travel fare of the path. The discomfort cost is not informed of in the developed algorithm. Before the journey, a passenger can decide travel path by considering whether the fare or time of the path the passenger would choice is smaller than that of other optional paths. We define this cost as the generalized cost function that can be represented as

(1) $$\begin{array}{c} C_{\text{arc}}=\alpha \cdot t_{f}\cdot t_{\text{arc}}+\beta \cdot f_{\text{arc}}, \end{array}$$

where *t*_*f*_ represents average values of time for the set of arcs and *α*, *β* denote, respectively, time parameter and fare parameter, *α* + *β* = 1.

As an example, consider the running arc (*v*_*a*,*T*1_^*s*,*de*^, *v*_*b*,*T*1_^*s*,*ar*^) as shown in Figure 1. Assume that the time of the departure node is 17:08 and the time of the arrival node is 18:34 and that the fare of this running arc is 24.5 CNY. Then, we can calculate the generalized cost of the running arc based on (1) as

(2) $$\begin{array}{c} C_{\text{arc}}=\alpha \times t_{f}\times \left(18:34-17:08\right)+\beta \times 24.5. \end{array}$$

In the railway passenger travel network, the generalized cost of the path is the sum of all arcs belonging to this path. Define the arc weight (*C*, *N*), where *C* and *N* represent the generalized cost and the residual capacity of arc, respectively. In particular, define the residual capacity of the boarding arc, stopping arc, transferring arc, and alighting arc *N* = *∞*, the generalized cost of boarding arc and alighting arc *C* = 0, and the fare of the stopping arc *f* = 0. The minimal capacity of all arcs in a path is the capacity of this path.

### 2.4. Network Topology Analysis Based on Residual Train Capacity

**Figure 3:**
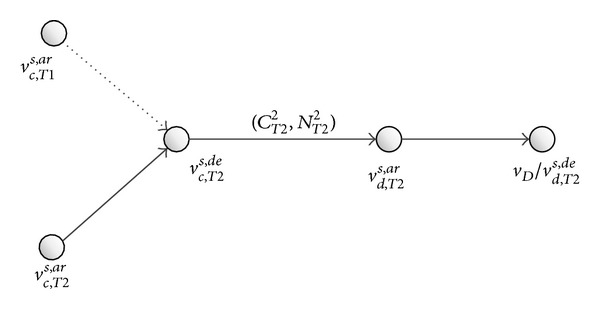
Network topology in condition that the train has residual capacity.

**Figure 4:**
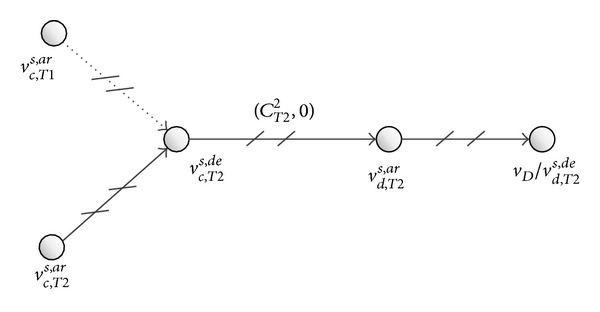
Network topology of disabled running arc.

Residual train capacity is declining when more and more passengers travel in the passenger travel network (Figure 3). In particular, the running arc of a train cannot transport more passengers when the train has no residual capacity; in other words, the running arc is disabled. Then the passenger travel network is changed. In Figure 4, the running arc (*v*_*c*,*T*2_^*de*^, *v*_*d*,*T*2_^*ar*^) is disabled when the train cannot transport more passengers; then the departure node *v*_*c*,*T*2_^*de*^ and the arrival node *v*_*d*,*T*2_^*ar*^ are disabled. According to network features, the stopping arc (*v*_*c*,*T*2_^*ar*^, *v*_*c*,*T*2_^*de*^) and the transferring arc (*v*_*c*,*T*1_^*ar*^, *v*_*c*,*T*2_^*de*^) which connected to the node *v*_*c*,*T*2_^*de*^ are disabled. Similarly, the stopping arc (*v*_*d*,*T*2_^*ar*^, *v*_*d*,*T*2_^*de*^) and the alighting arc (*v*_*d*,*T*2_^*ar*^, *v*_*D*_) which connected to the node *v*_*d*,*T*2_^*ar*^ are disabled.

### 2.5. Construction Algorithm of Passenger Travel Network Based on Train Timetable

The passenger travel network construction algorithm is shown in Algorithm 1.

## 3. Traveling Optimal Path Searching Algorithm

### 3.1. Analysis of the Optimal Paths Searching Problems

In this paper, we consider the following optimal path searching problem of railway passenger travel network: passenger flow guidance and passenger flow equilibrium distribution.

With regard to passenger flow guidance problem, passengers, whose travel time is definite, choose their optimal paths prior to their departure and then strictly follow their paths till they reach their destinations. They consider actual travel time, travel fare, or generalized cost in the actual train timetable (Table 1).

With regard to passenger flow equilibrium distribution problem, it refers to two situations including passenger flow distribution in emergence scenarios and simulation analysis of passenger flow distribution. In emergency scenarios, all stranded passengers need to be reasonably and efficiently guided to a safe place (another city). As for normal scenarios, the simulated passenger flow distribution is also required for the analogy and analysis of the practical passenger flow distribution.

Therefore, a general, feasible, and flexible method which could solve both the problems we revealed above is expected.

### 3.2. Traveling Optimal Path Searching Algorithm (TOPSA)

Search the optimal path by Dijkstra's algorithm and then assign passenger flow to this path according to the capacity of the optimal path until a running arc of this path is disabled. A new passenger travel network is formed after the disable arc is deleted. Continue to search the optimal path and assign the remaining passengers until all passengers are assigned or all paths have no residual capacity. TOPSA is shown in Algorithm 2.

### 3.3. Computational Complexity of TOPSA

Generally, the upper bound of the computational complexity of optimal paths search algorithm (Dijkstra's algorithm) from origin node to destination node is *O*(*n* ^2^), where *n* represents total number of nodes in the travel network. In TOPSA, in this paper, the algorithm stops when the remaining passengers *P* − ∑_*k*_ *N*_min⁡_(*l*_*k*_) ≤ 0. Therefore, the computational complexity of TOPSA is *O*(*k* · *n* ^2^), where *k* represents total number of optimal paths. In addition, the total number of optimal paths is limited by the size of the travel network and residual capacity; *k* (*k* ≪ *n*) is generally single digit. Thus, the computational complexity of TOPSA is *O*(*n* ^2^).

## 4. Numerical Example

**Figure 5:**
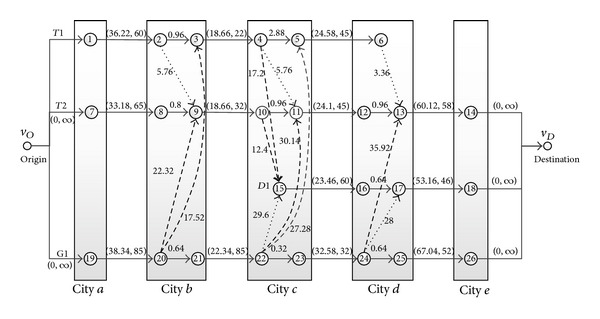
Railway passenger travel network based on railway timetables in the example.

The proposed approach is illustrated with a railway timetable as shown in Table 2. Four trains run among cities *a*, *b*, *c*, *d*, and *e*. Residual capacities of all trains are shown in Table 3. The minimal train transfer times of the same station and different station in a city are 15 min and 30 min, respectively. Use the proposed approach to construct railway passenger travel network based on the railway timetable (Table 2), as shown in Figure 5. Suppose all the fare of transferring arcs among different stations *f*_tarc_ = 30 CNY, the value of time *t*_*f*_ = 12 CNY/h, and the parameters *α* = 0.8 and *β* = 0.2.

In the travel network, suppose that 100 travelers need to travel from origin node of the city *a* to the destination node of the city *e*. The fare and capacity of running arcs are as shown in the Table 3. According to the method introduced in Section 3, four paths are found and passenger flow is assigned in the travel network, which are shown in Table 4, costing 0.46 s on the computer with an E7000+CPU and 2 G RAM.

Obviously, everyone hopes to choose the optimal/shortest path* P1* for his/her journey. However, in fact, the residual capacity of the train* T1* is not enough and only 32 travelers are distributed in the first path* P1*. Secondary optimal paths will be searched in a row and the remaining passengers will be distributed, respectively, until everyone can get on the train.

The number of passengers increases from 100 to the maximum capacity of trains, the optimal path is still searched, and the remaining passengers are assigned until all passengers are assigned or all paths have no residual capacity. For example, suppose that 250 travelers need to travel from origin node of the city *a* to the destination node of the city *e*. The fare and capacity of running arcs are unchanged. According to the method introduced in Section 3, five paths are found and passengers are assigned in the travel network, which are shown in Table 5, costing 0.50 s on the computer with an E7000+CPU and 2 G RAM. Only 136 travelers are assigned, and others cannot go to destination because all paths have no residual capacity in this travel network.

## 5. Conclusion

Railway transport enjoys great and increasing popularity as an essential transport mode for medium-long distance journey in many countries in the past decades. However, in condition when more and more different train types are available, railway passengers are often perplexed to choose the best travel plan. This study developed a rational approach to construct the railway passenger travel network, designed the generalized cost function on the path as the foundation of path searching procedure, and analyzed the network topology. In particular, we proposed an optimal path searching algorithm which combined the Dijkstra algorithm and the residual train capacity based on the railway passenger travel network, and the validity of the proposed approach was verified with an illustrative example. For future work, one possible extension of this research is to consider the transfer reliability and the path choice behaviors of various passenger groups. The proposed approach can push the construction of optimal train schedule and will be the theoretical foundation in decision making of resource allocation. In a more general sense of application, the proposed method could be adapted in multimodal transportation systems especially in metro transport.

## Figures and Tables

**Algorithm 1:**
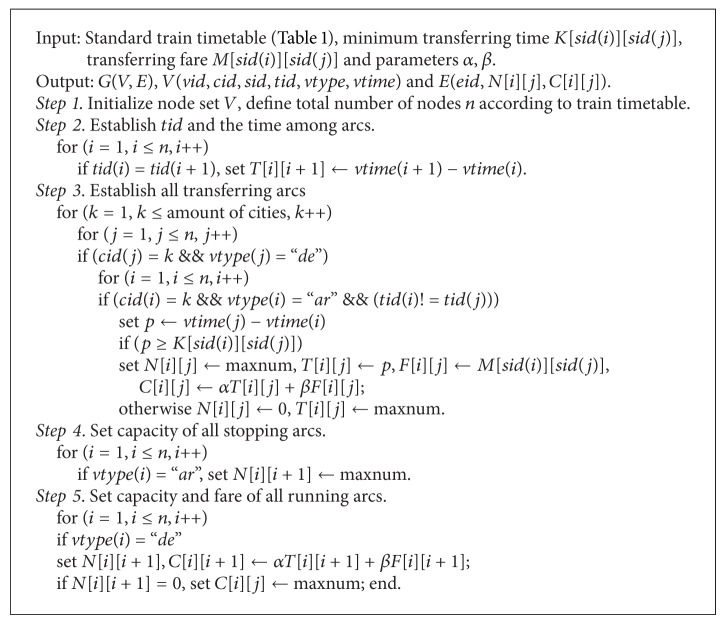


**Algorithm 2:**
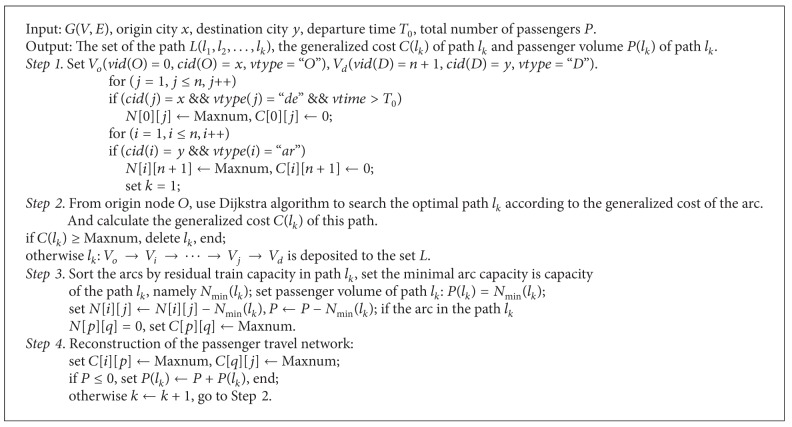


**Table 1:** Standard train timetable.

City	*a *				*b *		*c *			
Station	*a1 *		*a2 *		*b *		*c1 *		*c2 *	
Type	Arrival	Departure	Arrival	Departure	Arrival	Departure	Arrival	Departure	Arrival	Departure
Train										
* T1 *	—	—	—	17:08	18:34	18:52	20:35	—	—	—
* T2 *	—	15:00	—	—	17:33	17:38	—	—	20:50	20:56

**Table 2:** Railway timetable.

City	*a *		*b *		*c *		*d *		*e *	
Train	Arrival	Departure	Arrival	Departure	Arrival	Departure	Arrival	Departure	Arrival	Departure
*T1 *	—	14:10	17:02	17:08	18:34	18:52	20:35	—	—	—
*T2 *	—	15:00	17:33	17:38	19:04	19:10	20:50	20:56	01:38	—
*D1 *	—	—	—	—	—	19:44	20:40	20:44	22:50	—
*G1 *	—	14:37	15:56	16:00	16:39	16:41	17:49	17:53	19:47	—

**Table 3:** Fare (unit: CNY) and capacity of the running arcs among cities.

Arc	*a* → *b *		*b* → *c *		*c *→ *d *		*d* → *e *	
	Fare	Capacity	Fare	Capacity	Fare	Capacity	Fare	Capacity
*T1 *	43.5	60	24.5	22	40.5	45	—	—
*T2 *	43.5	65	24.5	32	40.5	45	75	58
*D1 *	—	—	—	—	72.5	60	165	46
*G1 *	128.5	85	80.5	85	108.5	32	244	52

**Table 4:** The optimal paths information for 100 travelers.

	Path	Generalized cost	Passenger flow
*P1 *	*v*_*o*_ → 7 → 8 → 9 → 10 → 11 → 12 → 13 → 14 → *v*_*D*_	138.78	32
*P2 *	*v*_*o*_ → 1 → 2 → 3 → 4 → 5 → 6 → 13 → 14 → *v*_*D*_	146.78	22
*P3 *	*v*_*o*_ → 19 → 20 → 21 → 22 → 23 → 24 → 25 → 26 → *v*_*D*_	161.90	32
*P4 *	*v*_*o*_ → 19 → 20 → 21 → 22 → 15 → 16 → 17 → 18 → *v*_*D*_	168.18	14

**Table 5:** The optimal paths information for 250 travelers.

	Path	Generalized cost	Passenger flow
*P1 *	*v*_*o*_ → 7 → 8 → 9 → 10 → 11 → 12 → 13 → 14 → *v*_*D*_	138.78	32
*P2 *	*v*_*o*_ → 1 → 2 → 3 → 4 → 5 → 6 → 13 → 14 → *v*_*D*_	146.78	22
*P3 *	*v*_*o*_ → 19 → 20 → 21 → 22 → 23 → 24 → 25 → 26 → *v*_*D*_	161.90	32
*P4 *	*v*_*o*_ → 19 → 20 → 21 → 22 → 15 → 16 → 17 → 18 → *v*_*D*_	168.18	46
*P5 *	*v*_*o*_ → 19 → 20 → 21 → 22 → 11 → 12 → 13 → 14 → *v*_*D*_	176.64	4
